# Retrorectal endometrioid cyst: a case report

**DOI:** 10.1186/1752-1947-4-389

**Published:** 2010-11-30

**Authors:** Iraklis E Katsoulis, Ioannis E Katsoulis

**Affiliations:** 1 White Cross Hospital, 1 Sisini Str, 11528 Athens, Greece

## Abstract

**Introduction:**

Developmental cysts are the most common retrorectal cystic lesions in adults, whereas reports of endometrioid cysts in this anatomic location are extremely rare.

**Case presentation:**

A 21-year-old nulliparous Greek woman presented with chronic noncyclic pelvic pain, and a retrorectal cyst was diagnosed. The lesion was resected through a laparotomy and, on histologic examination, was found to be an endometrioid cyst. The treatment was completed with a six-month course of a gonadotropin-releasing hormone analogue. One year after surgery, the woman remained free of symptoms, and pelvic imaging showed no recurrence of the lesion. Reviewing the literature, we found only three previous reports of an endometrioid cyst in this anatomic location.

**Conclusion:**

In women of reproductive age, endometriosis must be included in the differential diagnosis of retrorectal cysts.

## Introduction

Endometriosis is the presence of endometriotic tissue in anatomic regions outside the uterus [1]. The most-common sites are the ovaries and the fallopian tubes, the uterosacral ligaments, and the lateral pelvic peritoneum. Endometriosis can less commonly be found in laparotomy scars, the vagina, and the rectovaginal septum, and also can involve the wall of the colon and the rectum. This is a report of a rare retrorectal endometrioid cyst that was not contiguous to the rectal wall.

Developmental cysts are the most common retrorectal cystic lesions in adults, whereas reports of endometrioid cysts in this anatomic location are extremely rare [2-4].

## Case presentation

A 21-year-old nulliparous Greek woman complained of chronic noncyclic pelvic pain. Abdominal and vaginal examinations were unremarkable, whereas on rectal examination, a soft extraluminal mass was found posteriorly and left laterally.

**Figure 1:**
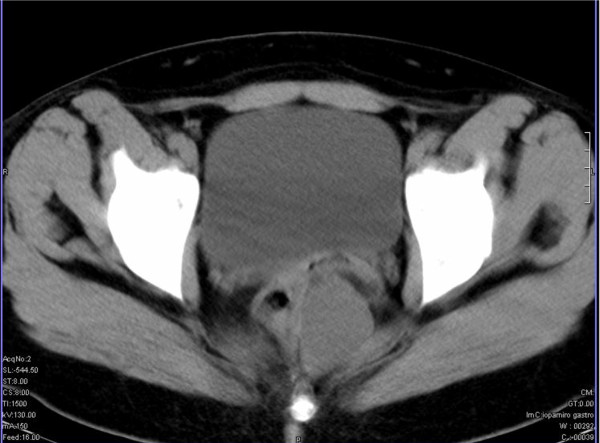
**Computed tomography, showing the cystic lesion posterior and left lateral to the middle rectum**.

The rectal mucosa was normal on rigid rectosigmoidoscopy. A pelvic ultrasound scan revealed a cystic lesion posterior to the middle rectum, and blood tests showed a moderately elevated CA 19-9 (79IU/ml), whereas all other tumour markers were normal. Computed tomography (CT) of the whole abdomen excluded other intra-abdominal pathology and provided further information regarding the anatomic relations of the lesion. The cyst lay posterior and left lateral to the middle rectum above the level of the pelvic floor and was contiguous neither to the rectal wall nor to the sacrum (Figure 1). Its maximal diameter was about 7 cm.

After administration of preoperative antibiotic prophylaxis, a laparotomy was undertaken through an infra-umbilical midline incision. Moderate bilateral ovarian endometriosis and minor endometriosis of the pelvic peritoneum were found; these were ablated with surgical diathermy. Subsequently, the pelvic peritoneum was opened, and the retrorectal space was carefully dissected to avoid injury of the pelvic nervous plexuses and the hypogastric nerves. The retrorectal cystic lesion was removed intact, and on histologic examination was found to be a suppurated endometrioid cyst.

The patient made an uneventful recovery and was discharged on the third postoperative day. The treatment was completed with a six-month course of a gonadotropin-releasing hormone (GnRH) analogue. One year postoperatively, she remained free of symptoms, and follow-up pelvic imaging showed no recurrence of endometriosis.

## Discussion

Developmental cysts are the most common retrorectal cystic lesions in adults, occurring mostly in middle-aged women. They are classified as epidermoid cysts, dermoid cysts, enteric cysts (tailgut cysts or hamartomas and cystic rectal duplication), and neuroenteric cysts, according to their origin and histopathologic features [5,6]. The diagnosis of retrorectal cysts can be accomplished with greater than 90% accuracy with computed tomography (CT) and magnetic resonance imaging (MRI) if the rectum is contrasted [3,6]. Such lesions warrant surgical excision to establish the diagnosis and to avoid complications. MRI has been suggested to increase the accuracy of preoperative localization and to enable surgical planning [6]. Transrectal ultrasound, if available, can also be useful in defining the depth of infiltration in cases of rectal involvement [3].

The operative approach can be perineal, abdominal, or combined, depending on the position of the lesion and its anatomic relations with surrounding structures. Retrorectal cysts have been also managed by using a laparoscopic approach [7]. In our patient, the information provided by the CT regarding the size and the anatomic relations of the cyst was considered sufficient, and therefore a pelvic MRI was not performed. We opted to approach the lesion through a laparotomy, aiming to explore her pelvis thoroughly in view of her persistent pelvic pain and elevated CA 19-9 levels.

We found foci of endometriosis on both ovaries and the pelvic peritoneum. A complete resection of the lesion was achieved, and histology made the diagnosis of a suppurated endometrioid cyst. In cases of low perirectal lesions, in which endometriosis is suspected, an alternative strategy can be transperineal excision combined with a laparoscopy for assessment of the intra-abdominal organs. We thought, however, that because the cyst lay posterior and left lateral to the middle rectum, a transperineal approach would neither be sufficient nor warrant the preservation of surrounding structures.

It is not uncommon for endometriosis to involve the rectal wall, requiring an anterior resection of the rectum [8]. Conversely, the presentation of an endometrioid cyst that occupies the retrorectal space, without being contiguous to either the rectal wall or the sacrum, is a rare entity. Reviewing the literature, we found only three previous reports of an endometrioid cyst in this anatomic location [2-4].

## Conclusion

In women of reproductive age, endometriosis must be included in the differential diagnosis of retrorectal cysts.

## Competing interests

The authors declare that they have no competing interests.

## Consent

Written informed consent was obtained from the patient for publication of this case report and accompanying images. A copy of the written consent is available for review by the Editor-in-Chief of this journal.

## Authors' contributions

Both authors contributed equally to the writing and read and approved the final manuscript.
